# Evaluating and Balancing the Risk of Breast Cancer-Specific Death and Other Cause-Specific Death in Elderly Breast Cancer Patients

**DOI:** 10.3389/fonc.2021.578880

**Published:** 2021-03-12

**Authors:** Yuan Peng, Taobo Hu, Lin Cheng, Fuzhong Tong, Yingming Cao, Peng Liu, Bo Zhou, Miao Liu, Hongjun Liu, Jiajia Guo, Fei Xie, Houpu Yang, Siyuan Wang, Chaobin Wang, Shu Wang

**Affiliations:** Breast Center, Peking University People's Hospital, Beijing, China

**Keywords:** balancing, other causes-specific death, breast cancer-specific death, elderly, breast cancer

## Abstract

**Purpose:** The dilemma of undertreatment and overtreatment of elderly breast cancer patients is common. This study aimed to investigate clinicopathological features, treatment modalities, and survival in women diagnosed with breast cancer at age 70 years or over, and to assist clinicians in developing individualized treatment plans by balancing the risks of breast cancer-specific death (BCSD) and other cause-specific death (OCSD).

**Methods:** This retrospective study included 420 women who were diagnosed with pathologically confirmed invasive breast cancer at age 70 years or older from January 2008 to December 2015 at Peking University People's Hospital (PKUPH). We collected baseline health status, tumor characteristics, treatment choices, and outcomes and created nomograms for clinicians to estimate individualized BCSD and OCSD risk directly.

**Results:** During a median follow-up of 71.5 months (range 2 to 144 months) in patients with stage I–III tumors, breast cancer specific survival (BCSS) was 92.4% (376/407) and overall survival (OS) was 78.1% (318/407). There were 89 deaths, and 65.2% (58/89) were non-breast cancer related. Upon multivariate analysis by Cox regression model, tumor size, positive lymph nodes, Ki-67, and surgery were independent predictors of BCSS, and comorbidities, positive lymph nodes, Ki-67, surgery, and endocrine therapy were independent predictors of OS. Propensity score weighted (PSW) was applied to analyze therapeutic efficacy, and there was BCSS and OS benefit with surgery (both *p* < 0.001), BCSS benefit with chemotherapy (*p* = 0.029), BCSS and OS benefit with endocrine therapy (*p* = 0.006 and 0.004), and neither BCSS nor OS benefit with radiotherapy (RT) (*p* = 0.348 and 0.289). Competing-risk nomograms were developed to estimate cumulative mortality probabilities for BCSD and OCSD for individual patients according to clinicopathologic characteristics and treatments. The calibration curves displayed exceptionally, with C-indexes 0.714 for BCSD and 0.717 for OCSD.

**Conclusions:** Older patients had greater risk of dying from non-breast cancer causes. Surgery, chemotherapy, and endocrine therapy were associated with improved survival. Competing risk nomograms allowed individual assessment of BCSD and OCSD, based on clinicopathological characteristics and treatment options, and can be used as a tool to help in choosing appropriate treatment strategies.

This study was approved by the Peking University People's Hospital Research Ethics Board on September 4, 2018.

## Introduction

Generally, because of concerns about the health status and treatment tolerance of elderly patients, doctors and patients are more likely to choose less than standard treatment (1). The impact of undertreatment on breast cancer specific survival (BCSS) among older patients remains controversial. Some studies indicate that rates of locoregional recurrence is not increased in comparison with conventionally treated elderly patients (2), while others show that undertreatment is associated with breast cancer death (3). On the other hand, age-related health problems in older patients decrease life expectancy and increase the risk of death from other causes other than breast cancer (4). This may reduce positive impact of standard treatment on the overall survival of elderly breast cancer patients. For elderly patients, it is important to balance the risks of breast cancer-specific death (BCSD) and other cause-specific death (OCSD). This makes the choice of treatment for elderly breast cancer patients more difficult.

The goal of this retrospective study was to examine baseline health status, clinicopathological characteristics, treatment course, and survival in women diagnosed with invasive breast cancer at age 70 years or older at our center, to assess the survival benefits of treatments using propensity score weighted (PSW) analysis, and to build a nomogram for clinicians to directly estimate individual cumulative incidences of BCSD and OCSD.

## Methods

### Patient Population

This retrospective study included 420 consecutive female patients from January 2008 to December 2015. The inclusion criteria to identify eligible patients were as follows: (1) female; (2) age 70 years or older at diagnosis; (3) pathologically confirmed invasive breast cancer by core-needle biopsy or excisional biopsy; Patients were excluded because of missing all information on baseline health status, tumor characteristics, treatment choices, and survival data. They accounted for 11.6% of 3,609 patients admitted in the same period. All patients had pathologically confirmed invasive breast cancer (core needle biopsy or surgery). Tumor size, lymph node status, hormone receptor (HR) status, HER-2 status, treatment [surgery, chemotherapy, endocrine therapy, radiotherapy (RT)], local-regional recurrence, metastasis, and survival were collected, along with baseline health data including body mass index (BMI), comorbidities, and activities of daily living (ADL) score. For this analysis, the definition of comorbidity was based on the Charlson Comorbidity Index (5), and the comorbidities still required medical treatment at the time of breast cancer diagnosis. The ADL score we used was basic activity of living (Barthel index). The scale described 10 tasks (including feeding, bathing, grooming, dressing, bowels, bladder, toilet use, transfer bed to chair and back, mobility on level surface and stairs). Total score was from 0 to 100, with lower scores indicating more loss of function. Follow-up was obtained from electronic chart review looking at the most recent medical record or from telephone follow-up with patient every 6 months after adjuvant therapy.

### Statistical Analysis

Disease-free survival (DFS) is defined as the time from diagnosis to recurrence, including locoregional disease or distant metastases, or to death from any cause. Breast cancer specific survival (BCSS) is defined as the time from diagnosis to death from breast cancer. Overall survival (OS) is defined as the time from diagnosis of breast cancer to death from any cause.

We evaluated the associations between course of treatment and clinicopathological features using *t*-tests, χ^2^-tests, and logistic regression models for multivariate analysis. Survival curves were estimated by Kaplan-Meier analysis. In the multivariate analysis, a Cox proportional hazards regression model was used to estimate whether a factor was a significant independent factor for survival. All of the above analyses were performed using the SPSS 20.0 software. Propensity score weighted (PSW) was applied using R 3.5.3 to eliminate clinicopathological and other treatment mixed bias and investigate the effect of chemotherapy, endocrine therapy, and local treatment on survival. When evaluating the impact of chemotherapy on survival, we balance all other factors (age, complications, tumor size, lymph node status, HR, HER2, Ki67, surgery or not, endocrine therapy or not and radiotherapy or not). When evaluating the impact of endocrine therapy on survival, we balance other factors (age, complications, tumor size, lymph node status, HER2, Ki67, surgery or not, chemotherapy therapy or not and radiotherapy or not). When evaluating the impact of radiotherapy on survival, we balance other factors (age, complications, tumor size, lymph node status, HR, HER2, Ki67, chemotherapy or not and endocrine therapy). When evaluating the impact of different local treatment on survival, we balance all other factors (age, complications, tumor size, lymph node status, HR, HER2, Ki67, endocrine therapy or not and chemotherapy or not).

We built nomograms to predict cumulative mortality probabilities of BCSD and OCSD for individual patients. With BCSD and OCSD as the competing endpoint events in the competing risk analysis, we used the R rms package to formulate the competing risk nomograms based on the coefficients from the Fine and Gray's model to predict 1-, 3-, and 5-year risk of BCSD and OCSD. During the validation process, concordance index (C-index) curves were chosen by using the R pec and DescTools packages. The C-index quantified the predictive ability of the model. The perfect prediction should fall on a 45-degree straight line passing through the origin. We performed bootstrapping with 1,000 resamples and 5-fold cross-validation.

## Results

### Patient Characteristics

A total of 420 women were included in the analysis. The median age at diagnosis of invasive breast cancer was 76 years (range 70–91 years), and 103 of the patients (24.5%) were aged 80 years or older. Nearly four-fifths (78.8%) of the patients had at least one comorbidity and nearly 40% had ADL scores <100. As shown in Table 1, the ADL scores decreased with age. Patients older than 80 years had a higher incidence of HER-2 negative tumors than those 70–79, but other tumor characteristics (stage, hormone status) were similar across age groups.

**Table 1:** Clinicopathologic and treatment characteristics of patients.

**Characteristics**	**Total (*****n*** **= 420)**		**Age 70–74 (*****n*** **= 173)**		**Age 75–79 (*****n*** **= 144)**		**Age ≥80 (*****n*** **= 103)**		
	**No. of patients**	**%**	**No. of patients**	**%**	**No. of patients**	**%**	**No. of patients**	**%**	***p***
Body mass index (BMI)									0.289
<24	158	37.6	60	34.7	51	35.4	47	45.6	
24–28	169	40.2	71	41.0	64	44.4	34	33.0	
≥28	93	22.1	42	24.3	29	20.1	22	21.4	
Number of comorbidities									0.536
0	89	21.2	38	22.0	34	23.6	17	16.5	
1–2	249	59.3	104	60.1	84	58.3	61	59.2	
≥3	82	19.5	31	17.9	26	18.1	25	24.3	
Activities of daily living score (ADL)^*^									0.001
100	154	60.4	74	73.3	54	58.7	26	41.9	
90–99	62	24.3	16	15.8	27	29.3	19	30.6	
≤89	39	15.3	11	10.9	11	12.0	17	27.4	
Tumor size									0.247
≤2 cm	271	64.5	118	68.2	93	64.6	60	58.3	
>2 cm	149	35.5	55	31.8	51	35.4	43	41.7	
Positive lymph nodes									0.273
0	267	63.6	112	64.7	86	59.6	69	67.0	
1–3 (N1)	91	21.7	35	20.2	31	21.5	25	24.3	
≥4 (N2-3)	62	14.8	26	15.0	27	18.8	9	8.7	
TNM stage									0.298
I	191	45.5	84	48.6	64	44.4	43	41.7	
II	151	36.0	60	34.7	50	34.7	41	39.8	
III	65	15.5	25	14.5	27	18.8	13	12.6	
IV	13	3.1	4	2.3	3	2.1	6	5.8	
Histology									0.847
Ductal	316	75.2	130	75.1	105	72.9	81	78.6	
Lobular	48	11.4	20	11.6	19	13.2	9	8.7	
Other	56	13.3	23	13.3	20	13.9	13	12.6	
Hormone receptor									0.365
Positive	330	78.6	134	77.5	110	76.4	86	83.5	
Negative	90	21.4	39	22.5	34	23.6	17	16.5	
HER-2^$^									0.013
Positive	39	10.3	18	11.5	19	14.3	2	2.3	
Negative	339	89.7	139	88.5	114	85.7	86	97.7	
Ki-67^#^									0.894
≤20%	255	61.3	103	60.2	87	61.3	65	63.1	
>20%	161	38.7	68	39.8	55	38.7	38	36.9	
Surgery									0.327
Breast conserving surgery	152	36.2	56	32.4	53	36.8	43	41.7	
Mastectomy	247	58.8	111	64.2	82	56.9	54	52.4	
No breast surgery	21	5.0	6	3.5	9	6.2	6	5.0	
Adjuvant chemotherapy									<0.001
Yes	147	35.0	77	44.5	51	35.4	19	18.4	
No	273	65.0	96	55.5	93	64.6	84	81.6	
Adjuvant radiation therapy^&^									0.012
Yes	61	17.7	38	24.1	17	14.4	6	8.8	
No	283	82.3	120	75.9	101	85.6	62	91.2	
Adjuvant endocrine therapy									0.136
Yes	332	79.0	136	78.6	108	75.0	88	85.4	
No	88	21.0	37	21.4	36	25.0	15	14.6	

* *ADL score was available for 255 patients*.

$ *HER-2 status was available for 378 patients*.

# *Ki-67 was available for 416 patients*.

& *Adjuvant radiation therapy was analyzed among patients (n = 344) received both breast and axillary surgery*.

### Treatment Options

**Figure 1:**
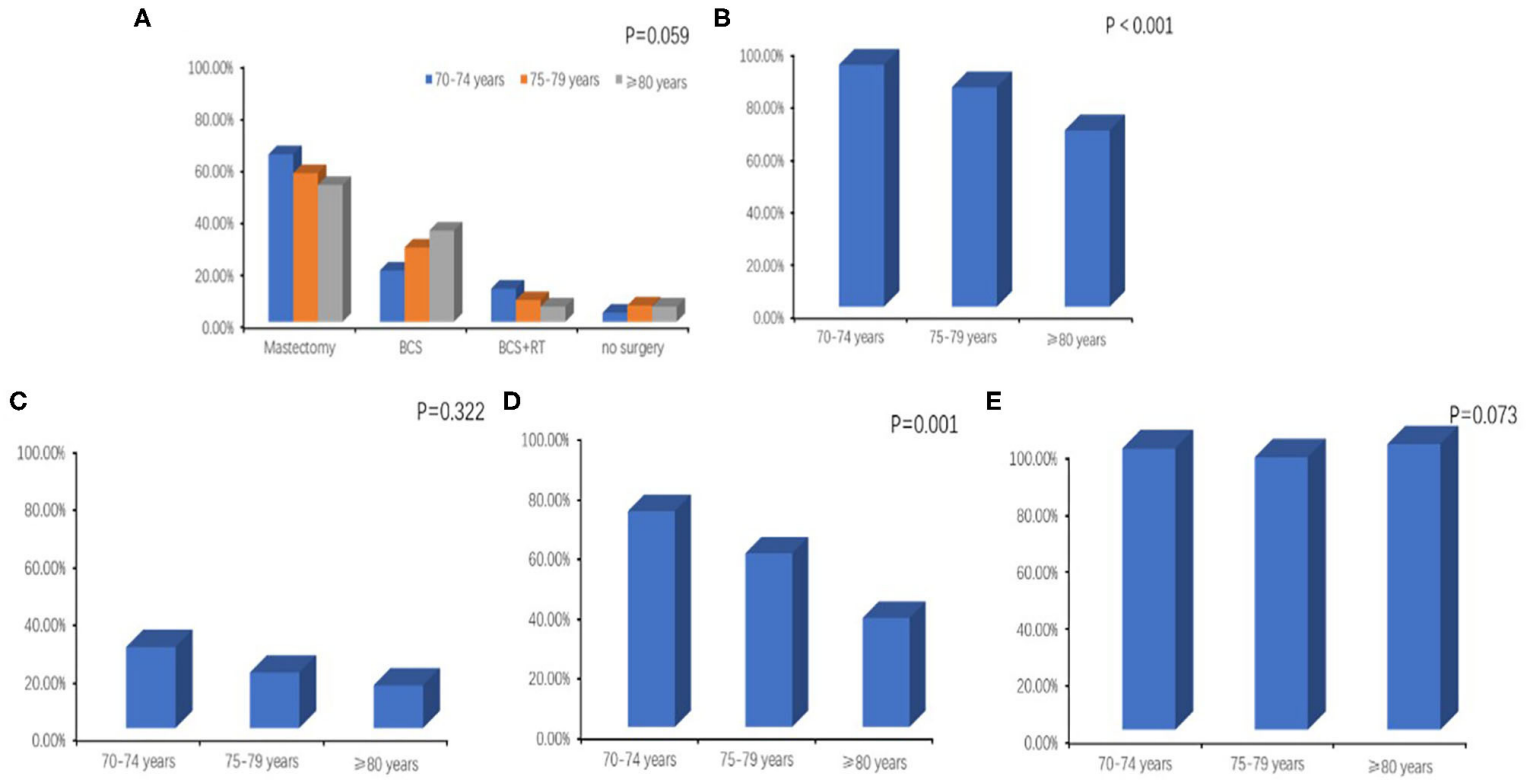
**(A)** Breast surgery with or without local radiotherapy by age. BCS, breast conserving surgery; RT, radiotherapy. **(B)** Axillary surgery (axillary lymph node dissection or sentinel lymph node biopsy) by age. **(C)** Regional lymph node radiotherapy by age. **(D)** Administration of chemotherapy according to age among patients with positive lymph nodes or triple-negative or HER-2 positive tumors. **(E)** Administration of endocrine therapy by age.

During the January 2008 to December 2015 study period, almost all of the included patients (399/420) had breast surgery, and 347 of the surgeries included axillary lymph node dissection (ALND) or sentinel lymph node biopsy (SLNB). Mastectomy was performed in 58.8% (247/420) of all patients, and the rate of mastectomy declined with age (64.2, 56.9, and 52.4% for ages 70–74, 75–79, and ≥80, respectively) while the incidence of breast conserving surgery (BCS) increased with age (19.7, 28.5, and 35.0%, ages 70–74, 75–79, and ≥80). Fewer patients in older age groups had BCS plus RT (12.7, 8.3, and 5.8% for patients 70–74, 75–79, and ≥80) (Figure 1A). Nearly all patients aged 70–74 had breast surgery with ALND or SLNB (91.9%), compared with 67.0% of patients aged 80 and above (Figure 1B). Among all patients with positive lymph nodes, 22.1% had regional lymph node RT, and the proportion was decreased with age, but not significantly (28.1, 19.2 vs. 14.8%, *p* = 0.322) (Figure 1C). The 2019 Guideline of the Chinese Society of Clinical Oncology (CSCO) recommends chemotherapy for breast cancers with positive lymph nodes and for triple-negative or HER-2 positive tumors. Among all 195 patients with the above, 114 (58.5%) received chemotherapy, and the number significantly decreased with age (72.0, 58.9 vs.36.4%, *p* = 0.001) (Figure 1D). Finally, endocrine therapy was prescribed in almost all patients with HR positive tumors (98.5, 95.5, and 100.0%, ages 70–74, 75–79, and ≥80) (Figure 1E).

There are many factors that might have affected treatment decisions for local treatment and chemotherapy in elderly patients for stage I–III patients (407/420). In univariate analysis, age, comorbidities, ADL sores, and clinical lymph node status were predictive of breast and axillary surgery, and after multivariate analysis, age, ADL score, and clinical lymph node status remained as independent factors. Age, comorbidities, ADL score, tumor size, positive lymph nodes, HR status, HER-2 status, and Ki-67 were meaningfully associated with chemotherapy in univariate analysis, and at multivariate analysis, age and ADL score remained significant, along with comorbidities, tumor size, positive lymph nodes, and HR status. Age, positive lymph nodes, and surgery method (mastectomy or BCS) were independently associated with RT (Supplementary Tables 1–6).

### Survival

The median follow-up was 71.5 months (range 2–144 months). At the time of analysis, DFS for stage I–III patients was 75.9% (309/407), OS was 78.1% (318/407), and BCSS was 92.4% (376/407). The OS for patients with stage IV tumors was 30.8%, and 77.8% (7/9) of deaths were breast cancer-related.

More than half of the patients (65.2%) who died during the study period died from non-breast cancer causes. The risk of dying from other causes was greater than the risk of dying from breast cancer at all ages. Among all patients who died, the proportion of deaths from other causes increased with advancing age but did not reach statistical significance (Table 2).

**Table 2:** Association between cause of death and age among women with I–III breast cancer.

**Characteristics**	**Age 70–74 (*****n*** **= 169)**		**Age 75–79 (*****n*** **= 141)**		**Age ≥80 (*****n*** **= 97)**		
	**No. of patients**	**%**	**No. of patients**	**%**	**No. of patients**	**%**	***p***
Status at end of follow-up period							0.022
Alive	143	84.6	106	75.2	69	71.1	
Dead	26	15.4	35	24.8	28	28.9	
Among women who died							0.384
Death due to breast cancer	11	42.3	13	35.3	7	25.0	
Death due to other causes	15	57.7	22	64.7	21	75.0	

**Figure 2:**
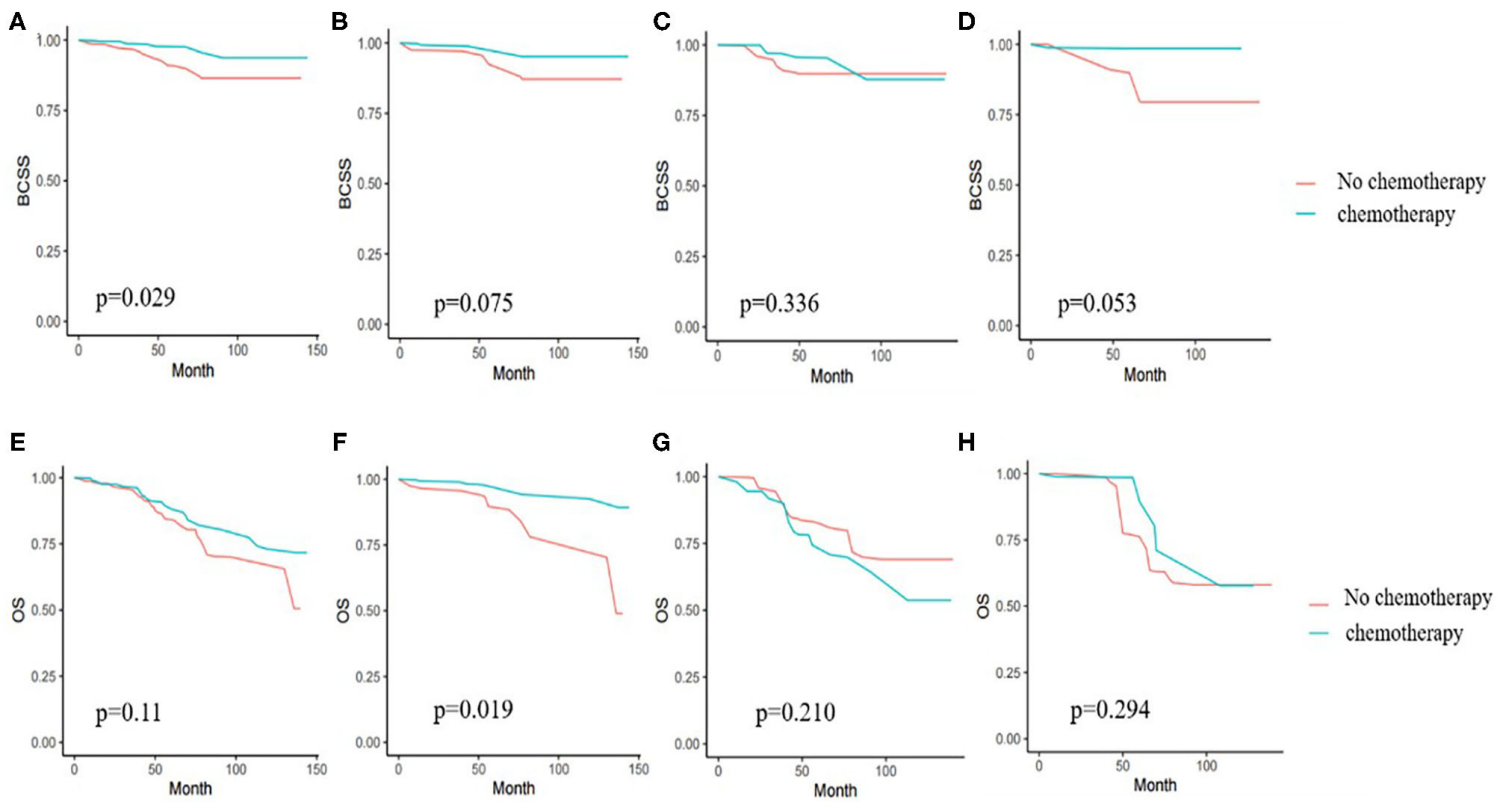
**(A)** Kaplan-Meier analyses of the effect of chemotherapy on breast cancer specific survival (BCSS) in weighted samples of all patients. **(B)** BCSS in patients with age from 70 to 74 years. **(C)** BCSS in patients with age from 75 to 79 years. **(D)** BCSS in patients age 80 years or older. **(E)** Kaplan-Meier analyses of the effect chemotherapy on overall survival (OS) in weighted samples. **(F)** OS in patients with age from 70 to 74 years. **(G)** OS in patients with age from 75 to 79 years. **(H)** OS in patients age 80 years or older.

**Figure 3:**
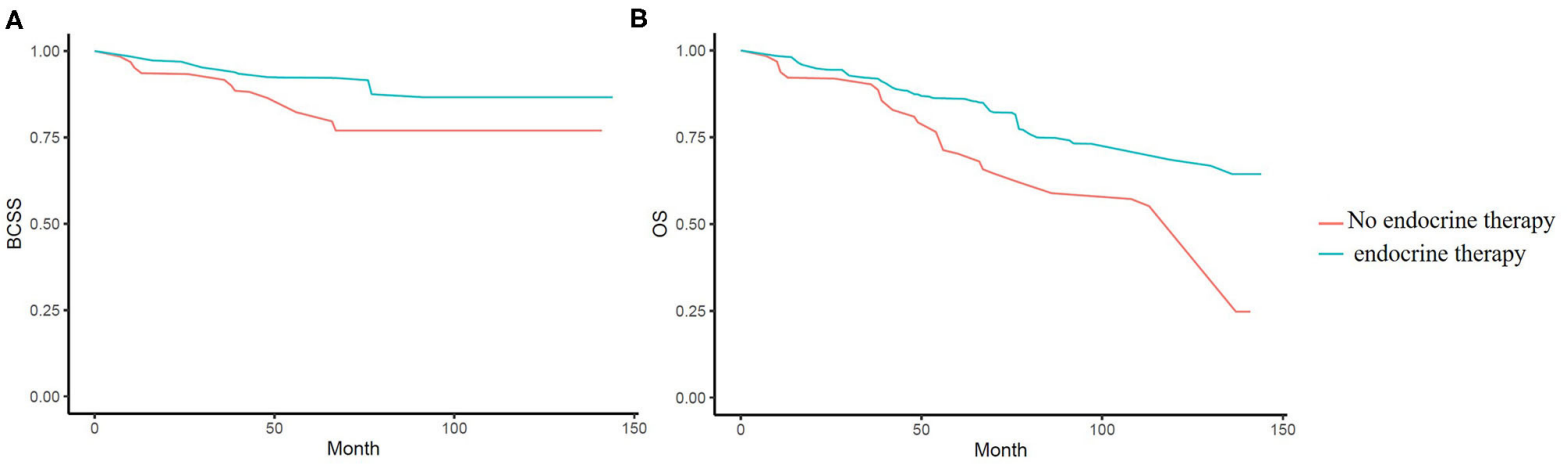
**(A)** Kaplan-Meier analyses of the effect of endocrine therapy on breast cancer specific survival (BCSS) in weighted samples. **(B)** Kaplan-Meier analyses of the effect of endocrine therapy on overall survival (OS) in weighted samples.

Kaplan-Meier survival analyses were performed in original samples of patients with stage I–III disease (Supplementary Tables 7, 8). We performed PSW to balance the influences of factors such as tumor characteristics, age, complications, and other therapies on the outcomes. Before weighted, the BCSS and OS of the chemotherapy group had no obviously difference than that of the no chemotherapy group (*p* = 0.411 and 0.994). After weighted, we saw a benefit from chemotherapy in BCSS (*p* = 0.029), but not in OS (*p* = 0.11). Since the interaction of age and chemotherapy was significant, we performed subgroup analyses and found that chemotherapy was associated with a significant reduction in overall mortality only in the 70–74-year age group and not in the patients aged 75 or older (Figure 2). After weighted, endocrine therapy was beneficial to BCSS (*p* = 0.006) and OS (*p* = 0.004) (Figure 3). There was no BCSS and OS benefit with radiotherapy (*p* = 0.348 and 0.289) (Supplementary Figure 1). (The data before and after weighted have been provided in Supplementary Tables 9–11, the Kaplan-Meier curve before PSW shown in Supplementary Figures 2–4).

**Figure 4:**
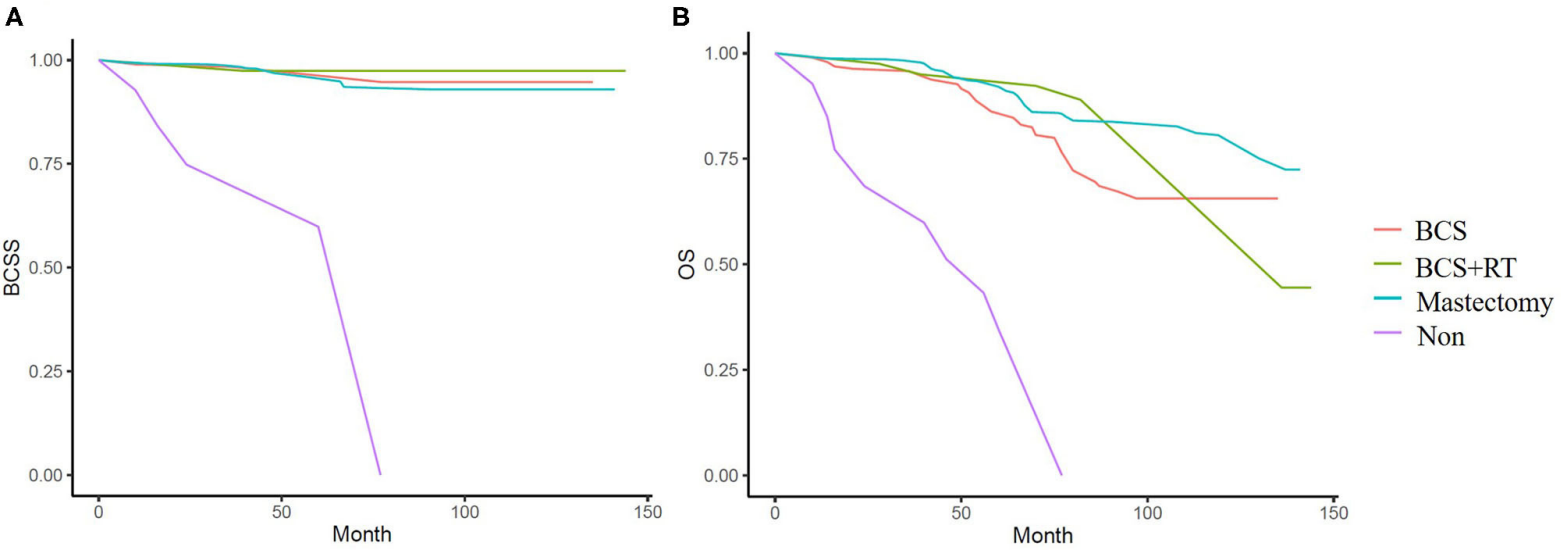
**(A)** Kaplan-Meier analyses of different local treatment on breast cancer specific survival (BCSS) in weighted samples. **(B)** Kaplan-Meier analyses of different local treatment on overall survival (OS) in weighted samples.

In the weighted samples, all types of local treatment (BCS+RT, BCS alone, and mastectomy) had equal BCSS and women who received no local treatment had a substantially increased risk of dying from breast cancer compared with those treated with surgery with/without RT (*p* < 0.001) (Figure 4A). In OS analysis, BCS+RT and mastectomy had equal survival (*p* = 0.456), and was better than BCS alone (*p* = 0.043 and 0.048), at the same time, they had obvious advantages compared with no local treatment (*p* < 0.001) (Figure 4B).

### Nomograms for BCSD and OCSD Prediction

**Figure 5:**
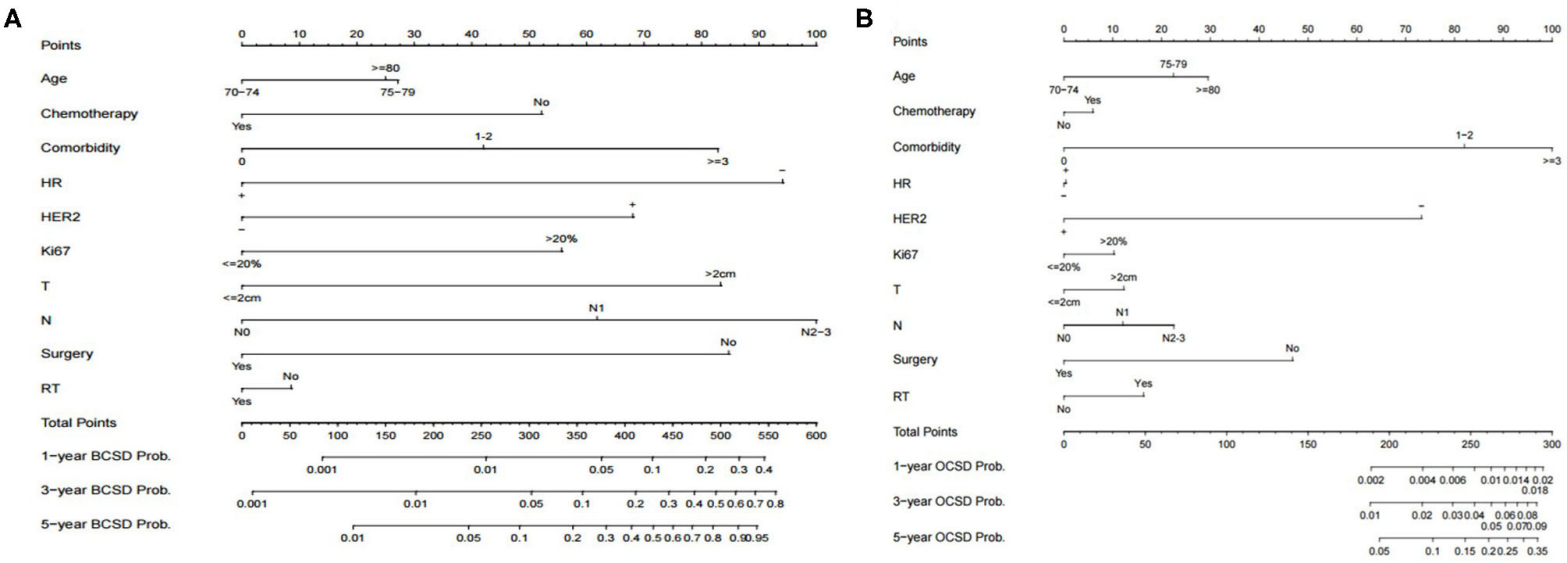
Competing risk nomograms predicting 1-, 3-, and 5-year cumulative probabilities for breast cancer-specific death (BCSD) and other cause-specific death (OCSD) in elderly women with breast cancer. **(A)** Breast cancer-specific death. **(B)** Other cause-specific death.

There is a substantial chance that elderly patients will die from non-breast cancer causes. In order to help clinicians make better treatment choices and allow patients to obtain overall survival benefits, we built predictive nomograms for BCSD and OCSD. As Figure 5 shows, a competing risk nomogram based on the Fine and Gray model was established to predict 1-, 3-, and 5-year cumulative death probabilities based on multivariate analysis. For each patient, her age, comorbidity, HR, HER2, Ki67, tumor size, lymph node stage, surgery or not, radiotherapy or not and chemotherapy or node have their own points. The predictive cumulative probabilities of BCSD and OCSD at 1-, 3-, and 5-years could be evaluated by the total score according to the bottom scale. For example, the 3-year BCSD was ~20% for patients age 70–74 years, with 1–2 comorbidities, T >2 cm, N1, HR negative, HER2 negative, Ki-67 >20%, received surgery, but no chemotherapy and radiotherapy.

**Figure 6:**
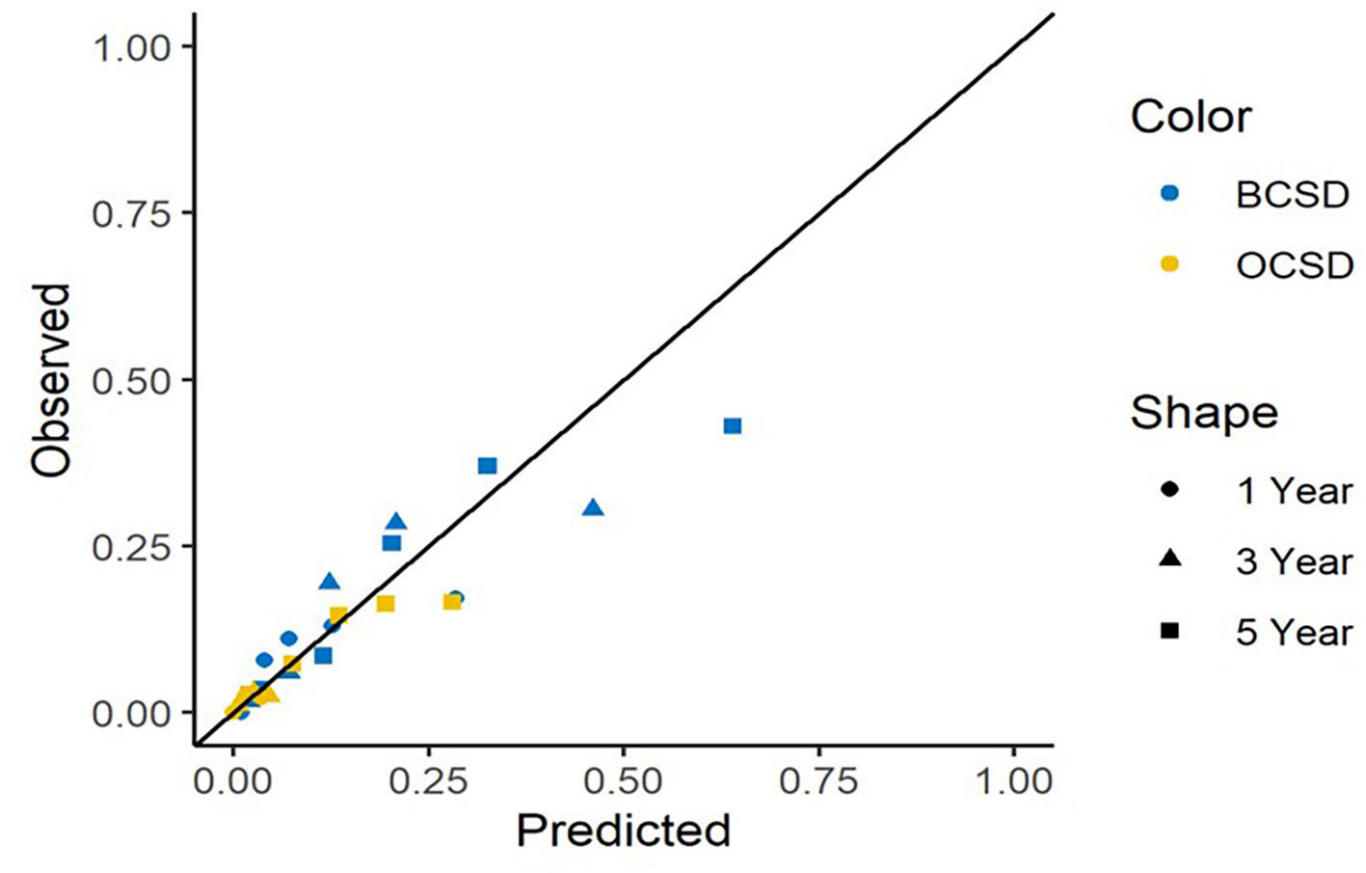
Calibration curves for 1-, 3-, and 5-year predictions.

The calibration curves for BCSD and OCSD are shown in Figure 6. All of the calibration curves appear to be close to the standard curves. For BCSD, the C-index score was 0.714 in the test cohort, demonstrating excellent predictive ability. For OCSD, the C-index was 0.717 in the test cohort. The competing risk nomogram performed well in internal validation.

## Discussion

Elderly patients are a special group of breast cancer patients, in the absence of evidence based standardized treatment, clinicians, and patients will make treatment choices based on their own experience and preferences (6). It is important to understand the tumor characteristics of elderly breast cancer patients well, to evaluate their physical conditions comprehensively, and to make treatment choices cautiously.

Treatment of elderly cancer patients is often complicated by a variety of chronic diseases, and it has been reported, for elderly patients, the probability of dying from other diseases, including cardiovascular disease, respiratory infection, diabetes mellitus, and others, is high; even >50% (7). This rate even reaches 80% in elderly patients with stage I breast cancer (8). In our study, 80% of the patients had at least one comorbidity, and 40% (101/255) had compromised ADLs, as indicated by the ADL scores, which decreased with age. During a median follow-up of 71.5 months, 65.2% of deaths were from non-breast cancer causes. This trend was more obvious with aging, and among the patients aged 80 years or older, 75% died from causes other than breast cancer. This makes the choice of systemic therapy complex, with the benefits of treatment to be balanced against a higher competing risk of death from causes other than cancer.

Competing-risk nomograms were developed to estimate cumulative mortality probabilities for BCSD and OCSD. As expected, tumor characteristics (i.e., HR-negative, HER-2 positive, and high Ki-67 expression) were as important as tumor stage in BCSD risk, but did not influence OCSD. The probability of both BCSD and OCSD increased slightly with aging. Comorbidities had a greater impact on OS than chronological age, and were the predominant driver of OCSD, as has been reported elsewhere (9, 10). Comorbidities also affected BCSD, which may because of the choice of less standard treatments for patients with more comorbidities.

For patients with early breast cancer, surgery is the most effective way to reduce BCSD without increasing the risk of OCSD. Previous evidence shows that surgery is almost always feasible for older patients, with outcomes comparable to younger groups and superior to non-surgical treatments (11). In our study, surgery was an independent factor to improve both BCSS and OS. Regardless of age, mastectomy was the most common local treatment for all patients, while BCS became more common with aging. After weighted, there was no significant difference in BCSS for patients receiving mastectomy, BCS, or BCS+RT, and all of them were better than no surgery. In our opinion, surgery should always be considered, regardless of age, if patient's physical condition allows. Mastectomy, BCS, and BCS+RT are all acceptable options, with multidisciplinary teamwork including oncologists and anesthesiologists, breast cancer surgery is mostly safe and effective.

The nomogram analysis also indicated that chemotherapy can significantly reduce the risk of BCSD and slightly increase the risk of OCSD. Elderly patients are less likely to receive adjuvant chemotherapy even when deemed appropriate candidates, and increasing age has been strongly associated with a decreasing likelihood of receiving chemotherapy (12, 13). At the same time, it has been shown that standard adjuvant chemotherapy was superior among older patients compared to compromised chemotherapy (14). In this cohort of elderly breast cancer patients, we demonstrated that chemotherapy varies by clinicopathological features at diagnosis (e.g., tumor size, lymph node metastasis, and HR status) and confirmed that age and comorbidity were associated with decreasing chemotherapy use. After weighted, we found that chemotherapy improved survival, especially in women aged 70–74 years, but not significantly in women 75 years or older. These results are similar to those in other studies that have shown benefit of chemotherapy in appropriately selected patients (15–18), but that the benefit decreases with aging (19, 20). At the same time, the physiologic changes of aging increase the risk of adverse effects, together with an increased risk of hospitalization (21). Chemotherapy may increase OCSD, although reported mortality from chemotherapy-related complications was low (22). Models for predicting chemotherapy toxicity in elderly patients, such as CRASH and CARG (23, 24), can assist in making treatment choices. As our results confirmed, after comprehensive assessment, in elderly patients with indications for chemotherapy, standard chemotherapy is still worthy.

Radiotherapy is quite controversial in the treatment of elderly patients. Schonberg et al. (19) found that older women treated with BCS+RT had the best breast cancer survival, while in the CALGB9343 (25) and PRIMEII (26) trials for elderly patients, recurrence risk was similar with or without RT in low risk patients. In our competing risk analysis, RT has no obvious effect on the survival in terms of BCSD and OCSD.

The competing risk nomogram for predicting BCSD and OCSD in elderly breast cancer patients can be readily applied in clinical practice. We can predict an older patient's prognosis according to her own characteristics. For example, RT need not be recommended for an 82-year-old woman with 5 comorbidities, already receiving surgical treatment, with a T1, N2, triple-negative, high Ki-67 tumor. If she decides not to receive regular chemotherapy, then from the nomogram, we can estimate that the 1-, 3-, and 5-year mortalities due to breast cancer are nearly 10, 20, and 40%. If she receives chemotherapy, the BCSD declines to 5, 10, and 20%. At the same time, consistent with the competing risk nomogram, we will predict that the 1-, 3-, and 5-year probabilities of OCSD are ~0.6%, 4%, and 15%, indicating that even at an advanced age and with many comorbidities, chemotherapy can still be strongly recommended.

There are several limitations in this study. Since this is a retrospective study, there is potential for selection bias, insufficient sample size, and missing data. We could not distinguish regional RT from whole-breast irradiation after breast-conserving surgery, so we had difficulty understanding the effect of regional RT on elderly patients. Endocrine therapy is an important treatment for elderly breast cancer patients, and an insufficient duration of endocrine therapy may affect the analysis of patient survival, which we cannot assess in this study. Nonetheless, although the current data is limited, it does not affect the competing risk nomogram model, which has good clinical application prospects. Because of the limitation of the number of patients and events, we just did internal validation after built the nomogram using the same group of patients. We are still recruiting patients prospectively for external validation, and more patients are needed to improve models in the future.

In conclusion, older patients had greater risk of dying from non-breast cancer causes. A competing risk nomogram based on clinicopathologic characteristics and treatment choices was built, and it can serve as a useful tool for balancing the risk of BCSD and OCSD and devising appropriate treatment strategies.

## Data Availability Statement

The original contributions presented in the study are included in the article/Supplementary Materials, further inquiries can be directed to the corresponding author.

## Ethics Statement

The studies involving human participants were reviewed and approved by Peking University People's Hospital Medical Ethics Committee. Written informed consent for participation was not required for this study in accordance with the national legislation and the institutional requirements.

## Author Contributions

YP and TH: methodology. LC, FT, YC, PL, BZ, ML, HL, JG, FX, HY, SW, and CW: resources. YP: data curation and writing—original draft preparation. SW: writing—review and editing. All authors have read and agreed to the published version of the manuscript.

## Conflict of Interest

The authors declare that the research was conducted in the absence of any commercial or financial relationships that could be construed as a potential conflict of interest.
