# Supplementary material for: Evaluating and Balancing the Risk of Breast Cancer-Specific Death and Other Cause-Specific Death in Elderly Breast Cancer Patients
Source: Front Oncol. 2021 Mar 12;11:578880. doi: 10.3389/fonc.2021.578880 (PMC7994517; doi:10.3389/fonc.2021.578880)
Supplement: Supplementary file 1 [file Data_Sheet_1.docx]

Supplementary Material

# Supplementary Tables

Supplementary Table 1. Factors associated with breast and axillary surgery on elderly breast cancer patients in univariate analysis

| Variable | Surgery (both breast and axillary) | | χ^2^ | P |
| --- | --- | --- | --- | --- |
|  | Yes(n=344) | No(n=63) |  |  |
| Age(years) |  |  | 25.881 | ＜0.001 |
| 70-74 | 158(93.5%) | 11(6.5%) |  |  |
| 75-79 | 118(83.7%) | 23(16.3%) |  |  |
| ≥80 | 68(70.1%) | 29(29.9%) |  |  |
| Comorbidities |  |  | 9.723 | 0.008 |
| 0 | 74(88.1%) | 10(11.9%) |  |  |
| 1-2 | 213(86.9%) | 32(13.1%) |  |  |
| ≥3 | 57(73.1%) | 21(26.9%) |  |  |
| ADL score | 95.3±8.705 | 88.0±18.557 |  | ＜0.001 |
| Clinical tumor size | | | 1.131 | 0.288 |
| ≤2cm | 226(85.9%) | 37(14.1%) |  |  |
| ＞2cm | 118(81.9%) | 26(18.1%) |  |  |
| Clinical lymph node | | | 5.833 | 0.016 |
| Negative | 238(81.8%) | 53(18.2%) |  |  |
| Positive | 106(91.4%) | 10(8.6%) |  |  |
| ER and/or PR |  |  | 3.502 | 0.061 |
| Positive | 264(82.8%) | 55(17.2%) |  |  |
| Negative | 80(90.9%) | 8(9.1%) |  |  |
| HER2 |  |  | 0.730 | 0.393 |
| Positive | 27(79.4%) | 7(20.6%) |  |  |
| Negative | 283(85.0%) | 50(15.0%) |  |  |
| Ki-67 |  |  | 0.981 | 0.322 |
| ≤20% | 209(83.3%) | 42(16.7%) |  |  |
| ＞20% | 133(86.9%) | 20(13.1%) |  |  |

- ^*^ADL score was available for 245 patients
- ^$^HER-2 status was available for 367 patients
- ^#^Ki-67 was available for 404 patients

Supplementary Table 2. Factors associated with breast and axillary surgery on elderly breast cancer patients in multivariate analysis

| Variable | β | *Wald* | *OR* | 95% *CI* | P |
| --- | --- | --- | --- | --- | --- |
| Age | -0.670 | 7.633 | 0.512 | 0.318-0.823 | 0.006 |
| Comorbidities | -0.025 | 0.007 | 0.976 | 0.538-1.769 | 0.935 |
| ADL score | 0.420 | 9.243 | 1.043 | 1.015-1.072 | 0.002 |
| Clinical lymph node | 1.171 | 4.662 | 3.227 | 1.114-9.344 | 0.031 |
| ER and/or PR status | 0.363 | 0.447 | 1.438 | 0.496-4.173 | 0.504 |

Supplementary Table 3. Factors associated with chemotherapy on elderly breast cancer patients in univariate analysis

| Variable | Chemotherapy | | χ^2^ | P |
| --- | --- | --- | --- | --- |
|  | Yes(n=139) | No(n=268) |  |  |
| Age(years) |  |  | 20.447 | ＜0.001 |
| 70-74 | 74(43.8%) | 95(56.2%) |  |  |
| 75-79 | 49(34.8%) | 92(65.2%) |  |  |
| ≥80 | 16(16.5%) | 81(83.5%) |  |  |
| Comorbidities |  |  | 7.914 | 0.019 |
| 0 | 37(44.0%) | 47(56.0%) |  |  |
| 1-2 | 84(34.3%) | 161(65.7%) |  |  |
| ≥3 | 18(23.1%) | 60(76.9%) |  |  |
| ADL score 92.9±12.489 97.6±4.879 | | |  | ＜0.001 |
| Tumor size | | | 16.014 | ＜0.001 |
| ≤2cm | 73(27.3%) | 194(72.7%) |  |  |
| ＞2cm | 66(47.1%) | 74(52.9%) |  |  |
| Positive Lymph node | | | 72.741 | ＜0.001 |
| 0 | 52(19.8%) | 211(80.2%) |  |  |
| 1-3(N1) | 44(52.4%) | 40(47.6%) |  |  |
| ≥4(N2-3) | 43(71.7%) | 17(28.3%) |  |  |
| ER and/or PR |  |  | 62.795 | ＜0.001 |
| Positive | 77(24.1%) | 242(75.9%) |  |  |
| Negative | 62(70.5%) | 26(29.5%) |  |  |
| HER2 |  |  | 13.058 | ＜0.001 |
| Positive | 21(61.8%) | 13(38.2%) |  |  |
| Negative | 100(30.0%) | 233(70.0%) |  |  |
| Ki-67 |  |  | 14.419 | ＜0.001 |
| ≤20% | 67(26.7%) | 184(73.3%) |  |  |
| ＞20% | 69(45.1%) | 84(54.9%) |  |  |

Supplementary Table 4. Factors associated with chemotherapy on elderly breast cancer patients in multivariate analysis

| Variable | β | | *Wald* | *OR* | 95% *CI* | P |
| --- | --- | --- | --- | --- | --- | --- |
| Age | | -0.880 | 7.478 | 0.415 | 0.221-0.779 | 0.006 |
| Comorbidities | -0.880 | | 6.019 | 0.415 | 0.206-0.838 | 0.014 |
| ADL score | 0.102 | | 9.129 | 1.107 | 1.036-1.182 | 0.003 |
| Tumor size | 1.067 | | 5.423 | 2.905 | 1.184-7.130 | 0.020 |
| Positive lymph nodes | 2.042 | | 33.305 | 7.703 | 3.851-15.409 | <0.001 |
| ER and/or PR status | 1.900 | | 11.809 | 6.684 | 2.262-19.751 | 0.001 |
| HER2 status | -0.538 | | 0.644 | 0.584 | 0.157-2.173 | 0.422 |
| Ki-67 | 0.392 | | 0.674 | 1.480 | 0.580-3.779 | 0.412 |

Supplementary Table 5. Factors associated with radiotherapy after the surgery （both breast and axillary lymph node, n=344 ）on elderly breast cancer patients in univariate analysis

| Variable | Radiotherapy | | χ^2^ | P |
| --- | --- | --- | --- | --- |
|  | Yes(n=61) | No(n=283) |  |  |
| Age(years) |  |  |  | 0.012 |
| 70-74 | 38(24.1%) | 120(75.9%) |  |  |
| 75-79 | 17(14.4%) | 101(85.6%) |  |  |
| ≥80 | 6(8.8%) | 62(91.2%) |  |  |
| Comorbidities |  |  |  | 0.296 |
| 0 | 14(18.9%) | 60(81.1%) |  |  |
| 1-2 | 41(19.2%) | 172(80.8%) |  |  |
| ≥3 | 6(10.5%) | 51(89.5%) |  |  |
| ADL score | 97.4±5.303 | 94.9±9.136 |  | 0.067 |
| Tumor size | | |  | 0.093 |
| ≤2cm | 45(19.9%) | 181(80.1%) |  |  |
| ＞2cm | 16(13.6%) | 102(86.4%) |  |  |
| Positive Lymph node | | |  | 0.006 |
| 0 | 31(14.6%) | 181(85.4%) |  |  |
| 1-3(N1) | 12(15.6%) | 65(84.4%) |  |  |
| ≥4(N2-3) | 18(32.7%) | 37(67.3%) |  |  |
| ER and/or PR |  |  |  | 0.950 |
| Positive | 47(17.8%) | 217(82.2%) |  |  |
| Negative | 14(17.5%) | 66(82.5%) |  |  |
| HER2 |  |  |  | 0.366 |
| Positive | 7(25.9%) | 20(74.1%) |  |  |
| Negative | 53(18.7%) | 230(81.3%) |  |  |
| Ki-67 |  |  |  | 0.711 |
| ≤20% | 36(17.2%) | 173(82.8%) |  |  |
| ＞20% | 25(18.8%) | 108(81.2%) |  |  |
| Surgery |  |  |  | ＜0.001 |
| Mastectomy | 23(10.0%) | 208(90.0%) |  |  |
| BCS | 38(33.6%) | 75(66.4%) |  |  |

Supplementary Table 6. Factors associated with radiotherapy on elderly breast cancer patients in multivariate analysis

| Variable | β | *Wald* | *OR* | 95% *CI* | P |
| --- | --- | --- | --- | --- | --- |
| Age | -0.792 | 11.601 | 0.453 | 0.287-0.714 | 0.001 |
| Positive lymph node | 1.144 | 22.978 | 3.139 | 1.966-5.011 | ＜0.001 |
| Breast surgery | -2.434 | 36.930 | 0.088 | 0.040-0.192 | ＜0.001 |

Supplementary Table 7. Factors associated with breast cancer specific survival (BCSS) and overall survival (OS) in elderly breast cancer patients (Kaplan-Meier survival analyses) with original samples

| Variable | BCSS | | OS | |
| --- | --- | --- | --- | --- |
|  | Percent | P | Percent | P |
| Age (years) |  | 0.495 |  | 0.007 |
| 70-74 | 93.5% |  | 84.6% |  |
| 75-79 | 90.8% |  | 75.2% |  |
| ≥80 | 92.8% |  | 71.1% |  |
| Comorbidities |  | 0.029 |  | <0.001 |
| 0 | 91.7% |  | 89.3% |  |
| 1-2 | 94.7% |  | 78.8% |  |
| ≥3 | 85.9% |  | 64.1% |  |
| Tuner size |  | 0.001 |  | 0.003 |
| ≤2cm | 95.5% |  | 82.4% |  |
| >2cm | 86.4% |  | 70.0% |  |
| Positive lymph node |  | <0.001 |  | <0.001 |
| 0 | 95.4% |  | 83.3% |  |
| 1-3 (N1) | 90.5% |  | 71.4% |  |
| ≥4 (N2-3) | 81.7% |  | 65.0% |  |
| Hormone receptor |  | <0.001 |  | 0.009 |
| Positive | 95.0% |  | 80.9% |  |
| Negative | 83.0% |  | 68.2% |  |
| HER-2 |  | <0.001 |  | 0.003 |
| Positive | 73.5% |  | 67.6% |  |
| Negative | 94.0% |  | 79.6% |  |
| Ki-67 |  | <0.001 |  | 0.005 |
| ≤20% | 96.0% |  | 82.9% |  |
| >20% | 86.3% |  | 70.6% |  |
| Surgery |  | 0.007 |  | <0.001 |
| Yes | 93.6% |  | 81.4% |  |
| No | 85.7% |  | 60.3% |  |
| Chemotherapy |  | 0.411 |  | 0.994 |
| Yes | 90.6% |  | 76.3% |  |
| No | 93.3% |  | 79.1% |  |
| Endocrine therapy |  | <0.001 |  | 0.006 |
| Yes | 94.7% |  | 81.2% |  |
| No | 83.7% |  | 66.3% |  |
| Radiotherapy |  | 0.595 |  | 0.170 |
| Yes | 93.7% |  | 82.5% |  |
| No | 92.5% |  | 78.4% |  |

Supplementary Table 8(A). Multivariate analysis by Cox regression model to identify significant predictors of breast cancer specific survival with original samples

| Predictor | β | *Wald* | *HR* | 95% *CI* | P |
| --- | --- | --- | --- | --- | --- |
| Comorbidities | 0.461 | 2.318 | 1.586 | 0.876-2.870 | 0.128 |
| Tumor size | 0.906 | 5.169 | 2.473 | 1.133-5.399 | 0.023 |
| Positive lymph node | 0.683 | 8.368 | 1.981 | 1.247-3.147 | 0.004 |
| HR status | 1.184 | 1.900 | 3.269 | 0.607-17.613 | 0.168 |
| HER-2 status | -0.786 | 2.679 | 0.456 | 0.178-1.168 | 0.102 |
| Ki-67% | 1.100 | 6.690 | 3.005 | 1.305-6.917 | 0.010 |
| Surgery | -1.652 | 12.417 | 0.192 | 0.077-0.480 | <0.001 |
| Endocrine therapy | 0.533 | 0.393 | 1.705 | 0.321-9.045 | 0.531 |

Supplementary Table 8(B). Multivariate analysis by Cox regression model to identify significant predictors of overall survival with original samples

| Predictor | β | *Wald* | *HR* | 95% *CI* | P |
| --- | --- | --- | --- | --- | --- |
| Age | 0.211 | 1.711 | 1.235 | 0.900-1.694 | 0.191 |
| Comorbidities | 0.574 | 8.772 | 1.775 | 1.214-2.596 | 0.003 |
| Tumor size | 0.387 | 2.603 | 1.472 | 0.920-2.354 | 0.107 |
| Positive lymph node | 0.514 | 11.013 | 1.672 | 1.234-2.265 | 0.001 |
| HR status | -0.822 | 1.926 | 0.440 | 0.138-1.403 | 0.165 |
| HER-2 status | -0.349 | 0.807 | 0.705 | 0.329-1.511 | 0.369 |
| Ki-67% | 0.498 | 4.102 | 1.645 | 1.016-2.663 | 0.043 |
| Surgery | -1.308 | 19.422 | 0.270 | 0.151-0.484 | <0.001 |
| Endocrine therapy | -1.279 | 4.839 | 0.278 | 0.089-0.870 | 0.028 |

Supplementary Table 9 Data before and after PSW between chemotherapy or not

| Variable | Original samples | | P | Weighted samples | | P |
| --- | --- | --- | --- | --- | --- | --- |
|  | Chemotherapy | No chemotherapy |  | Chemotherapy | No chemotherapy |  |
| Age(years) |  |  | ＜0.001 |  |  | 0.973 |
| 70-74 | 74(53.2%) | 95(35.4%) |  | 69.4(49.9%) | 131.4(49.0%) |  |
| 75-79 | 49(35.3%) | 92(34.3%) |  | 47.3(34.0%) | 94.3(35.2%) |  |
| ≥80 | 16(11.5%) | 81(30.2%) |  | 22.3(16.0%) | 42.3(15.8%) |  |
| Comorbidities |  |  | 0.019 |  |  | 0.522 |
| 0 | 37(26.6%) | 47(17.5%) |  | 28.6(20.6%) | 63.1(23.5%) |  |
| 1-2 | 84(60.4%) | 161(60.1%) |  | 89.7(64.5%) | 157.4(58.7%) |  |
| ≥3 | 18(12.9%) | 60(22.4%) |  | 20.7(14.9%) | 47.5(17.7%) |  |
| Tumor size | | | ＜0.001 |  |  | 1.000 |
| ≤2cm | 73(52.5%) | 194(72.4%) |  | 80.7(58.1%) | 156.0(58.2%) |  |
| ＞2cm | 66(47.5%) | 74(27.6%) |  | 58.3(41.9%) | 112.0(41.8%) |  |
| Lymph node | | | ＜0.001 |  |  | 0.461 |
| Negative | 52(37.4%) | 211(78.7%) |  | 74.8(53.8%) | 156.0(58.2%) |  |
| Positive | 87(62.6%) | 57(21.3%) |  | 64.2(46.2%) | 112.0(41.8%) |  |
| ER and/or PR |  |  | ＜0.001 |  |  | 0.778 |
| Positive | 77(55.4%) | 242(90.3%) |  | 101.9(73.3%) | 201.3(75.1%) |  |
| Negative | 62(44.6%) | 26(9.7%) |  | 37.2(26.8%) | 66.8(24.9%) |  |
| HER2 |  |  | ＜0.001 |  |  | 1.000 |
| Positive | 21(61.8%) | 13(38.2%) |  | 12.3(10.2%) | 23.8(9.7%) |  |
| Negative | 100(30.0%) | 233(70.0%) |  | 108.7(89.8%) | 222.2(90.3%) |  |
| Ki-67 |  |  | ＜0.001 |  |  | 1.000 |
| ≤20% | 67(26.7%) | 184(73.3%) |  | 78.5(57.7%) | 154.6(57.7%) |  |
| ＞20% | 69(45.1%) | 84(54.9%) |  | 57.5(42.3%) | 113.4(42.3%) |  |
| Surgery |  |  | ＜0.001 |  |  | 0.994 |
| Yes | 133(95.7%) | 211(78.7%) |  | 132.1(95.0%) | 256.2(95.6%) |  |
| No | 6(4.3%) | 57(21.3%) |  | 6.9(5.0%) | 11.8(4.4%) |  |
| Radiotherapy |  |  | 0.018 |  |  | 1.000 |
| Yes | 30(21.9%) | 33(12.7%) |  | 22.8(16.6%) | 43.6(16.8%) |  |
| No | 107(78.1%) | 226(87.3%) |  | 114.2(83.4%) | 215.4(83.2%) |  |
| Endocrine therapy |  |  | ＜0.001 |  |  | 0.828 |
| Yes | 76(55.1%) | 244(91.0%) |  | 99.5(72.1%) | 197.4(73.7%) |  |
| No | 62(44.9%) | 24(9.0%) |  | 38.5(27.9%) | 70.6(26.3%) |  |

Supplementary Table 10 Data before and after PSW between endocrine therapy or not

| Variable | Original samples | | P | Weighted samples | | P |
| --- | --- | --- | --- | --- | --- | --- |
|  | Endocrine | No endocrine |  | Endocrine | No endocrine |  |
| Age(years) |  |  | 0.147 |  |  | 0.332 |
| 70-74 | 131(40.9%) | 37(43.0%) |  | 149.8(46.8%) | 36.8(42.8%) |  |
| 75-79 | 106(33.1%) | 35(30.7%) |  | 103.8(32.4%) | 35.0(40.7%) |  |
| ≥80 | 83(25.9%) | 14(16.3%) |  | 66.4(20.8%) | 14.2(16.5%) |  |
| Comorbidities |  |  | 0.612 |  |  | 0.791 |
| 0 | 63(19.7%) | 21(24.4%) |  | 77.9(24.3%) | 18.0(20.9%) |  |
| 1-2 | 194(60.6%) | 50(58.1%) |  | 180.2(56.3%) | 51.4(59.8%) |  |
| ≥3 | 63(19.7%) | 15(17.4%) |  | 62.1(19.4%) | 16.5(19.2%) |  |
| Tumor size | | | 0.008 |  |  | 0.928 |
| ≤2cm | 220(68.8%) | 46(53.5%) |  | 179.4(56.1%) | 49.3(57.3%) |  |
| ＞2cm | 100(31.2%) | 40(46.5%) |  | 140.7(43.9%) | 36.7(42.7%) |  |
| Lymph node | | | 0.001 |  |  | 0.970 |
| Negative | 218(68.1%) | 45(52.3%) |  | 163.1(51.0%) | 44.7(52.0%) |  |
| Positive | 102(31.9%) | 41(47.7%) |  | 157.0(49.0%) | 41.3(48.0%) |  |
| HER2 |  |  | ＜0.001 |  |  | 1.000 |
| Positive | 14(4.8%) | 20(26.7%) |  | 62.2(21.4%) | 15.8(21.1%) |  |
| Negative | 277(95.2%) | 55(73.3%) |  | 228.8(78.6%) | 59.2(78.9%) |  |
| Ki-67 |  |  | ＜0.001 |  |  | 1.000 |
| ≤20% | 229(71.6%) | 22(26.5%) |  | 107.8(33.7%) | 27.6(33.3%) |  |
| ＞20% | 91(28.4%) | 61(73.5%) |  | 212.2(66.3%) | 55.4(66.7%) |  |
| Surgery |  |  | 0.145 |  |  | 0.966 |
| Yes | 266(83.1%) | 77(89.5%) |  | 286.1(89.4%) | 76.1(98.5%) |  |
| No | 54(16.9%) | 9(10.5%) |  | 34.0(10.6%) | 9.9(11.5%) |  |
| Radiotherapy |  |  | 0.894 |  |  | 0.860 |
| Yes | 50(16.1%) | 13(15.5%) |  | 47.9(15.4%) | 14.2(16.9%) |  |
| No | 261(83.9%) | 71(84.5%) |  | 263.2(84.6%) | 69.8(83.1%) |  |
| Chemotherapy |  |  | ＜0.001 |  |  | 0.831 |
| Yes | 76(23.8%) | 62(72.1%) |  | 196.4(61.4%) | 54.5(63.4%) |  |
| No | 244(76.2%) | 24(27.9%) |  | 123.7(38.6%) | 31.5(36.6%) |  |

Supplementary Table 11 Data before and after PSW between radiotherapy or not

| Variable | Original samples | | P | Weighted samples | | P |
| --- | --- | --- | --- | --- | --- | --- |
|  | Radiotherapy | No radiotherapy |  | Radiotherapy | No radiotherapy |  |
| Age(years) |  |  | 0.001 |  |  | 0.798 |
| 70-74 | 39(61.9%) | 127(38.1%) |  | 39(61.9%) | 199.7(60.0%) |  |
| 75-79 | 17(27.0%) | 119(35.7%) |  | 17(27.0%) | 102.3(30.7%) |  |
| ≥80 | 7(11.1%) | 87(26.1%) |  | 7(11.1%) | 31.1(9.3%) |  |
| Comorbidities |  |  | 0.109 |  |  | 0.164 |
| 0 | 14(22.2%) | 70(21.0%) |  | 14(22.2%) | 97.4(29.2%) |  |
| 1-2 | 43(68.3%) | 194(58.3%) |  | 43(68.3%) | 185.1(55.6%) |  |
| ≥3 | 6(9.5%) | 69(20.7%) |  | 6(9.5%) | 50.7(15.2%) |  |
| Tumor size | | | 0.226 |  |  | 1.000 |
| ≤2cm | 46(73.0%) | 217(65.2%) |  | 46(73.0%) | 246.0(73.9%) |  |
| ＞2cm | 17(27.0%) | 116(34.8%) |  | 17(27.0%) | 86.8(26.1%) |  |
| Lymph node | | | 0.001 |  |  | 1.000 |
| Negative | 33(52.4%) | 227(68.2%) |  | 33(52.4%) | 165.4(49.7%) |  |
| Positive | 30(47.6%) | 106(31.8%) |  | 30(47.6%) | 167.8(50.4%) |  |
| ER and/or PR |  |  | 0.985 |  |  | 1.000 |
| Positive | 49(77.8%) | 260(78.1%) |  | 49(77.8%) | 256.8(77.1%) |  |
| Negative | 14(22.2%) | 73(21.9%) |  | 14(22.2%) | 76.2(22.9%) |  |
| HER2 |  |  | 0.486 |  |  | 1.000 |
| Positive | 7(11.3%) | 25(8.5%) |  | 7.9(12.7%) | 40.0(13.6%) |  |
| Negative | 55(88.7%) | 269(91.5%) |  | 54.1(87.2%) | 254.2(86.4%) |  |
| Ki-67 |  |  | 0.787 |  |  | 1.000 |
| ≤20% | 38(60.3%) | 205(62.1%) |  | 38(60.3%) | 199.2(60.3%) |  |
| ＞20% | 25(39.7%) | 125(37.9%) |  | 25(39.7%) | 131.0(39.7%) |  |
| Endocrine therapy |  |  | 0.894 |  |  | 1.000 |
| Yes | 50(79.4%) | 261(78.6%) |  | 50(79.4%) | 261.8(78.9%) |  |
| No | 13(20.6%) | 71(21.4%) |  | 13(20.6%) | 70.2(21.1%) |  |
| Chemotherapy |  |  | 0.018 |  |  | 1.000 |
| Yes | 30(47.6%) | 107(32.1%) |  | 30(47.6%) | 158.5(47.6%) |  |
| No | 33(52.4%) | 226(67.9%) |  | 33(52.4%) | 174.7(52.4%) |  |

Supplementary Table 12 Data before and after PSW between different local treatment

| Variable | Original samples | | | | P | Weighted samples | | | | P |
| --- | --- | --- | --- | --- | --- | --- | --- | --- | --- | --- |
|  | Mastectomy | BCS+RT | BCS | Non |  | Mastectomy | BCS+RT | BCS | Non |  |
| Age(years) |  |  |  |  | 0.018 |  |  |  |  | 0.140 |
| 70-74 | 110(45.6%) | 22(55.0%) | 34(30.6%) | 3(20.0%) |  | 103.3(42.9%) | 22(55.0%) | 37.7(34.0%) | 3.03(20.2%) |  |
| 75-79 | 81(33.6%) | 12(30.0%) | 41(36.9%) | 7(46.7%) |  | 75.1(31.2%) | 12(30.0%) | 43.3(39.0%) | 6.93(46.2%) |  |
| ≥80 | 50(20.7%) | 6(15.0%) | 36(32.4%) | 5(33.3%) |  | 62.5(25.9%) | 6(15.0%) | 30.0(27.0%) | 5.05(33.7%) |  |
| Comorbidities |  |  |  |  | 0.008 |  |  |  |  | 0.224 |
| 0 | 55(22.8%) | 10(25.0%) | 17(15.4%) | 2(13.3%) |  | 44.5(18.5%) | 10(25.0%) | 19.2(17.3%) | 2.0(13.5%) |  |
| 1-2 | 151(62.7%) | 26(65.0%) | 60(54.1%) | 8(53.3%) |  | 156.2(64.8%) | 26(65.0%) | 64.8(58.4%) | 7.9(52.9%) |  |
| ≥3 | 35(14.5%) | 4(10.0%) | 34(30.6%) | 5(33.3%) |  | 40.3(16.7%) | 4(10.0%) | 27.0(24.3%) | 5.1(33.7%) |  |
| Tumor size | | |  |  | <0.001 |  |  |  |  | 0.055 |
| ≤2cm | 137(56.8%) | 33(82.5%) | 90(81.1%) | 7(46.7%) |  | 181(75.1%) | 33(82.5%) | 87.4(78.7%) | 7.1(47.3) |  |
| ＞2cm | 104(43.2%) | 7(17.5%) | 21(18.9%) | 8(53.3%) |  | 60(24.9%) | 7(17.5%) | 23.6(21.3%) | 7.9(52.7) |  |
| Lymph node | | |  |  | <0.001 |  |  |  |  | <0.001 |
| Negative | 132(54.8%) | 28(70.0%) | 99(89.2%) | 4(26.7%) |  | 180.9(75.1%) | 28(70.0%) | 97.4(87.7%) | 4.04(26.9%) |  |
| Positive | 109(45.2%) | 12(30.0%) | 12(10.8%) | 11(73.3%) |  | 60.1(24.9%) | 12(30.0%) | 13.6(12.3%) | 10.96(73.1%) |  |
| ER and/or PR |  |  |  |  | 0.100 |  |  |  |  | 0.923 |
| Positive | 179(74.3%) | 33(82.5%) | 95(85.6%) | 12(80.0%) |  | 198.7(82.4%) | 33(82.5%) | 94.3(85.0%) | 12(80.0%) |  |
| Negative | 62(25.7%) | 7(17.5%) | 16(14.4%) | 3(20.0%) |  | 42.3(17.6%) | 7(17.5%) | 16.7(15.0%) | 3(20.0%) |  |
| HER2 |  |  |  |  | 0.082 |  |  |  |  | 0.067 |
| Positive | 21(9.8%) | 3(7.7%) | 6(6.0%) | 4(28.6%) |  | 17.5(8.2%) | 3.9(10%) | 6.2(6.2%) | 3.6(25.7%) |  |
| Negative | 193(90.2%) | 36(92.3%) | 94(94.0%) | 10(71.4%) |  | 196.5(91.8%) | 35.1(90.0%) | 93.8(93.8%) | 10.4(74.3%) |  |
| Ki-67 |  |  |  |  | 0.218 |  |  |  |  | 0.946 |
| ≤20% | 138(58.0%) | 26(65.0%) | 77(69.4%) | 10(66.7%) |  | 158.6(66.6%) | 26(65.0%) | 76.6(69.0%) | 10(66.7%) |  |
| ＞20% | 100(42.0%) | 14(35.0%) | 34(30.6%) | 5(33.3%) |  | 79.4(33.4%) | 14(35.0%) | 34.4(31.0%) | 5(33.3%) |  |
| Endocrine therapy |  |  |  |  | 0.161 |  |  |  |  | 0.719 |
| Yes | 181(75.4%) | 34(85.0%) | 94(84.7%) | 11(73.3%) |  | 198.9(82.5%) | 34(85.0%) | 93.5(84.2%) | 11(73.3%) |  |
| No | 59(24.6%) | 6(15.0%) | 17(15.4%) | 4(26.7%) |  | 13(17.5%) | 6(15.0%) | 17.5(15.8%) | 4(26.7%) |  |
| Chemotherapy |  |  |  |  | <0.001 |  |  |  |  | 0.532 |
| Yes | 107(44.4%) | 10(25.0%) | 18(16.2%) | 4(26.7%) |  | 58.8(24.4%) | 10(25.0%) | 19.8(17.8%) | 4(26.7%) |  |
| No | 134(55.6%) | 30(75.0%) | 93(83.8%) | 11(73.3%) |  | 33(75.6%) | 30(75.0%) | 91.2(82.8%) | 11(73.3%) |  |

Supplementary Table 13

R code for Propensity Score Weighting in this study

# load required libraries

library(readxl)

library(finalfit)

library(mice)

library(tableone)

library(Matching)

library(survey)

library(reshape2)

library(ggplot2)

# load data for modeling as 'rhc', replace incomplete cases

rhc <- read_excel('data.xlsx', col_types = "numeric")

rhc %>% mice() -> df

rhc <- complete(df)

# build the model for binominal logistic regression

psModel <- glm(formula = chemo ~ age + com + T + N + ER + Her2 + Ki67 + surgery + endo + radio,

family = binomial(link = "logit"),

data = rhc,

na.action = NULL)

rhc$pRhc <- predict(psModel, type = "response")

rhc$pNoRhc <- 1 - rhc$pRhc

rhc$pAssign <- NA

rhc$pAssign[rhc$chemo == 1] <- rhc$pRhc[rhc$chemo == 1]

rhc$pAssign[rhc$chemo == 0] <- rhc$pNoRhc[rhc$chemo == 0]

rhc$pMin <- pmin(rhc$pRhc, rhc$pNoRhc)

# calculate the weight

rhc$weight <- rhc$pMin / rhc$pAssign

write.csv(rhc, file = 'result/rhc.csv')

# build the model for multinominal logistic regression

test<-multinom(group~age + com +T+N+HR+Her2+Ki67+chemo+endo, data = rhc1)

prob<-predict(test, type='probs')

dim(prob)

rhc1$pRhc<- NA

rhc1$pRhc[rhc1$group == 1] <- prob[,1][rhc1$group == 1]

rhc1$pRhc[rhc1$group == 2] <- prob[,2][rhc1$group == 2]

rhc1$pRhc[rhc1$group == 3] <- prob[,3][rhc1$group == 3]

rhc1$pRhc[rhc1$group == 4] <- prob[,4][rhc1$group == 4]

rhc1$pNoRhc <- 1 - rhc1$pRhc

rhc1$pAssign <- NA

rhc1$pAssign[rhc1$group == 1] <- prob[,1][rhc1$group == 1]

rhc1$pAssign[rhc1$group == 2] <- prob[,2][rhc1$group == 2]

rhc1$pAssign[rhc1$group == 3] <- prob[,3][rhc1$group == 3]

rhc1$pAssign[rhc1$group == 4] <- prob[,4][rhc1$group == 4]

rhc1$pMin <- pmin(rhc1$pRhc, rhc1$pNoRhc)

rhc1$weight <- rhc1$pMin / rhc1$pAssign

R code for Nomogram in this study

# BCSS

mod <- cph(Surv(Tstart, Tstop, status == 1) ~ Age + Chemo + Com + HR + Her2 + Ki67 + Pt + Pn

+surgery+RT,

data = df.w,

weights = weight.cens,

subset = failcode == 1,

surv = T)

surv <- Survival(mod)

nom.sur <- nomogram(mod,

fun = list(function(x) 1 - surv(12, x),

function(x) 1 - surv(36, x),

function(x) 1 - surv(60, x)),

funlabel = c("1-year BCSS Prob.",

"3-year BCSS Prob.",

"5-year BCSS Prob."),

lp = F)

pdf("result/BCSS_nomogram.pdf", height = 9, width = 10)

plot(nom.sur,

fun.side = list(c(1, 1, 1, 3, 1, 3, 1, 3, 1, 3, 1, 3, 1, 3),

rep(1, 8),

rep(1, 10)))

dev.off()

# NBCSS

mod <- cph(Surv(Tstart, Tstop, status == 2) ~ Age + Chemo + Com + HR + Her2 + Ki67 + Pt + Pn + surgery+RT,

data = df.w,

weights = weight.cens,

subset = failcode == 2,

surv = T)

surv <- Survival(mod)

nom.sur <- nomogram(mod,

fun = list(function(x) 1 - surv(12, x),

function(x) 1 - surv(36, x),

function(x) 1 - surv(60, x)),

funlabel = c("1-year NBCSS Prob.",

"3-year NBCSS Prob.",

"5-year NBCSS Prob."),

lp = F)

pdf("result/NBCSS_nomogram.pdf", height = 9, width = 10)

plot(nom.sur,

fun.side = list(c(1, 1, 1, 3, 1, 3, 1, 3, 1, 3),

rep(1, 8),

rep(1, 10)))

dev.off()

# Supplementary Figures

Supplement Figure 1. (A) Kaplan-Meier analyses of the effect of radiotherapy on breast cancer specific survival (BCSS) in matched samples. (B) Kaplan-Meier analyses of the effect of radiotherapy on overall survival (OS) in matched samples.

Supplement Figure 2. (A) Kaplan-Meier analyses of the effect of chemotherapy on breast cancer specific survival (BCSS) in original samples of all patients. (B) Kaplan-Meier analyses of the effect chemotherapy on overall survival (OS) in original samples.

Supplement Figure 3. (A) Kaplan-Meier analyses of the effect of endocrine therapy on breast cancer specific survival (BCSS) in original samples. (B) Kaplan-Meier analyses of the effect of endocrine therapy on overall survival (OS) in original samples.

Supplement Figure 4. (A) Kaplan-Meier analyses of the effect of radiotherapy on breast cancer specific survival (BCSS) in original samples. (B) Kaplan-Meier analyses of the effect of radiotherapy on overall survival (OS) in original samples.

Supplement Figure 5. (A) Kaplan-Meier analyses of the effect of different local treatments on breast cancer specific survival (BCSS) in original samples. (B) Kaplan-Meier analyses of the effect of different local treatments on overall survival (OS) in original samples.
