# Supplementary figures and images for: Evaluating and Balancing the Risk of Breast Cancer-Specific Death and Other Cause-Specific Death in Elderly Breast Cancer Patients
Source: Front Oncol. 2021 Mar 12;11:578880. doi: 10.3389/fonc.2021.578880 (PMC7994517; doi:10.3389/fonc.2021.578880)

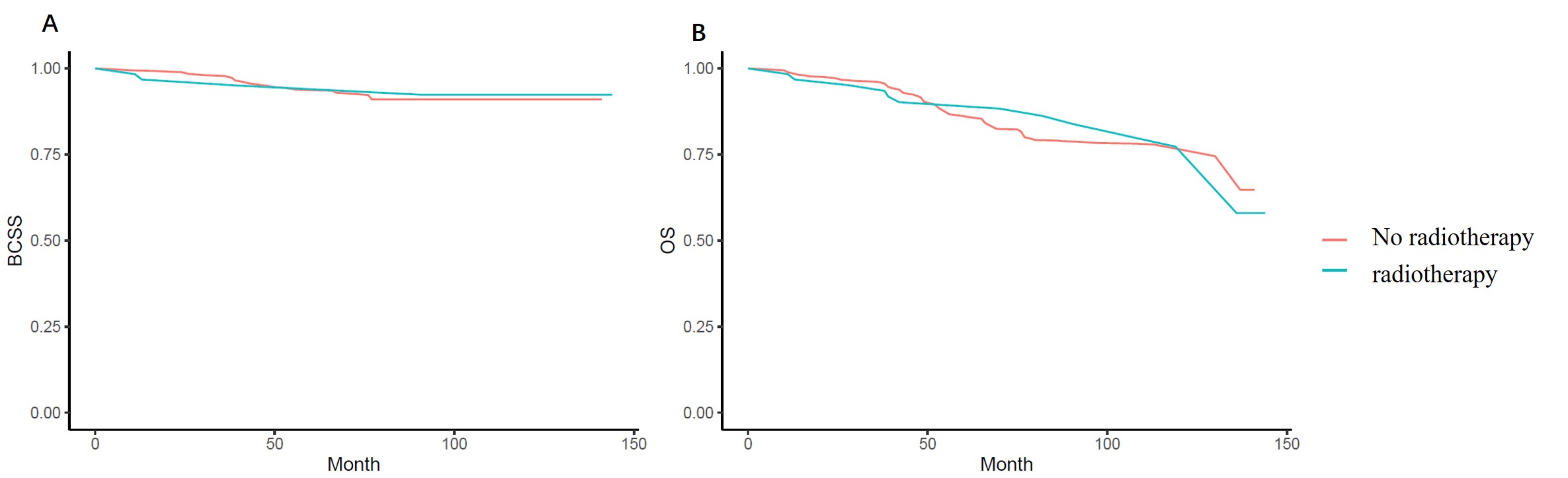

Supplement: Supplementary file 2 [file Image_1.JPEG]

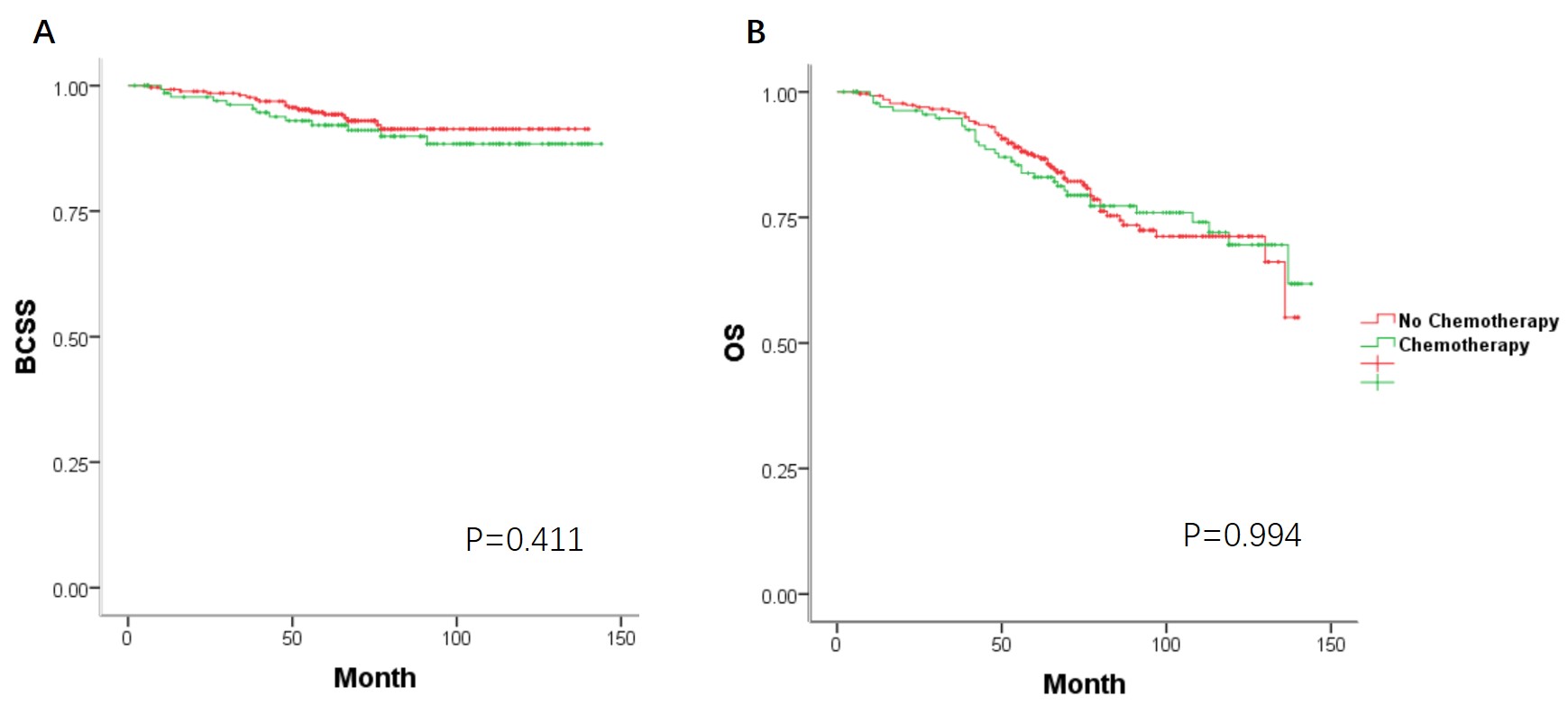

Supplement: Supplementary file 3 [file Image_2.JPEG]

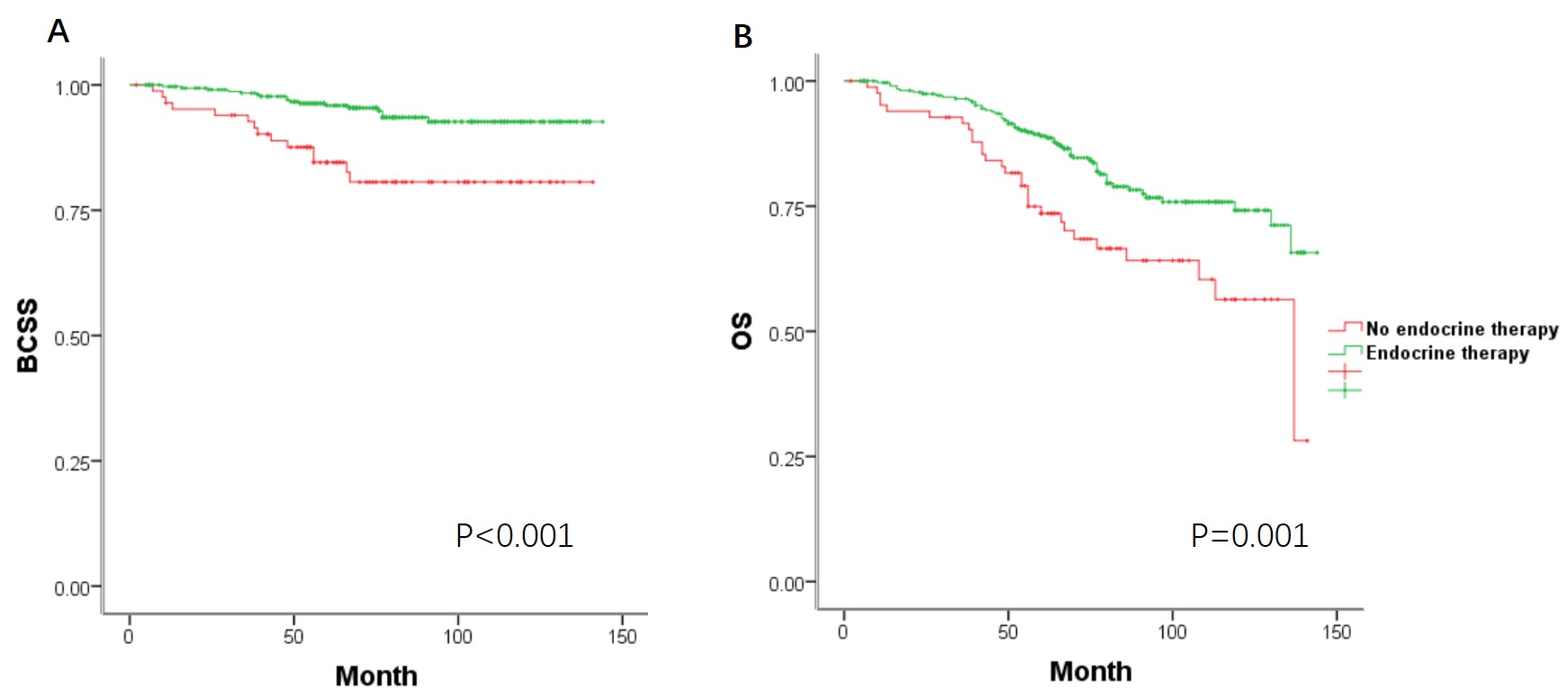

Supplement: Supplementary file 4 [file Image_3.JPEG]

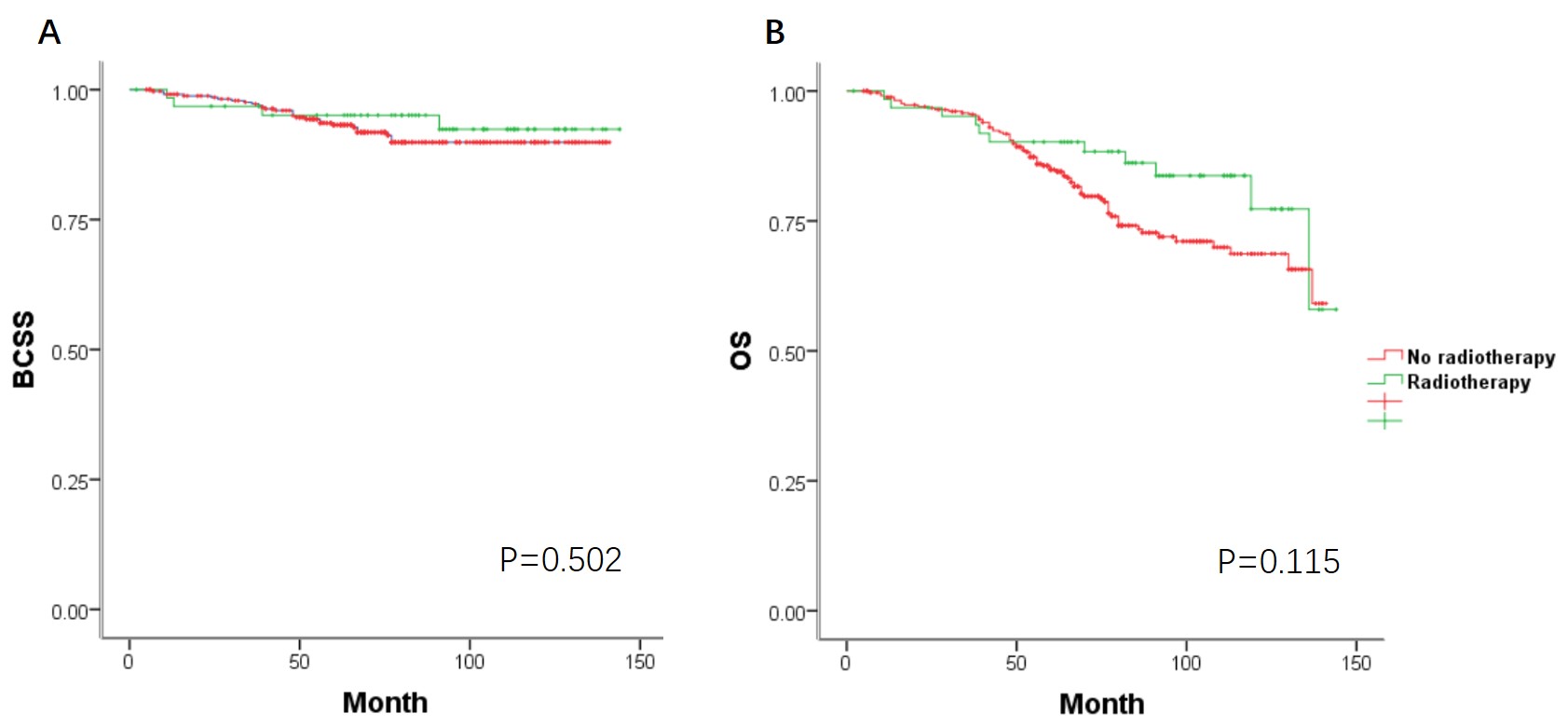

Supplement: Supplementary file 5 [file Image_4.JPEG]

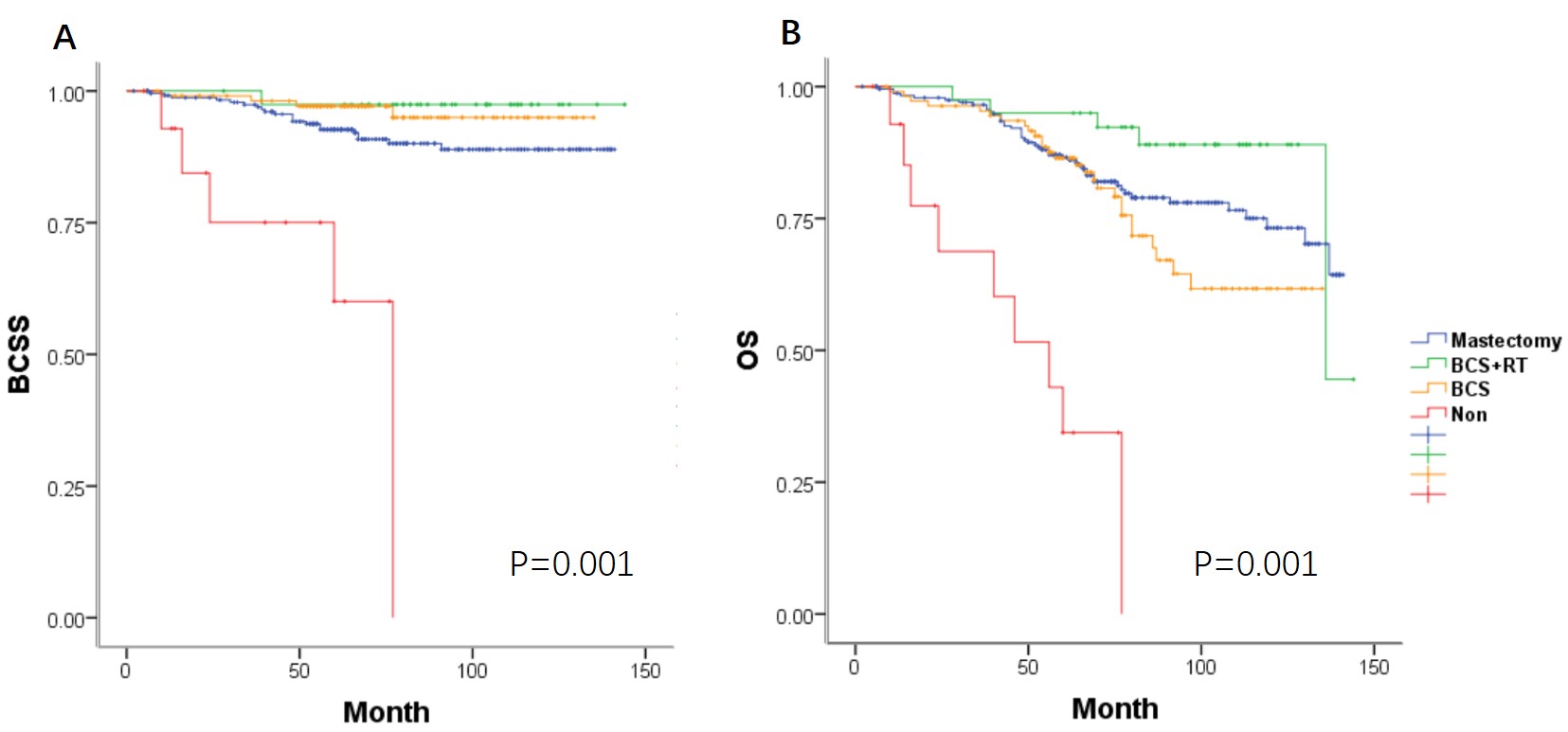

Supplement: Supplementary file 6 [file Image_5.JPEG]
